# Reduction in Irradiation Dose in Aperture Coded Enhanced Computed Tomography Imager Using Super-Resolution Techniques

**DOI:** 10.3390/s20226551

**Published:** 2020-11-16

**Authors:** Yossef Danan, Doron Avraham, Zeev Zalevsky

**Affiliations:** 1 LensFree Ltd., Ra’anana 4366241, Israel; yossefda@jce.ac.il (Y.D.); zeev.zalevsky@biu.ac.il (Z.Z.); 2 Faculty of Engineering, Bar-Ilan University, Ramat-Gan 52900, Israel

**Keywords:** biomedical imaging, super-resolution, coded aperture imaging, computed tomography

## Abstract

One of the main concerns regarding medical imaging is the danger tissue’s ionizing due to the applied radiation. Many medical procedures are based on this ionizing radiation (such as X-rays and Gamma radiation). This radiation allows the physician to perform diagnosis inside the human body. Still, the main concern is stochastic effects to the DNA, particularly the cause of cancer. The radiation dose endangers not only the patient but also the medical staff, who might be close to the patient and be exposed to this dangerous radiation in a daily manner. This paper presents a novel concept of radiation reduced Computed Tomography (CT) scans. The proposed concept includes two main methods: minification to enhance the energy concertation per pixel and subpixel resolution enhancement, using shifted images, to preserve resolution. The proposed process uses several pinhole masks as the base of the imaging modality. The proposed concept was validated numerically and experimentally and has demonstrated the capability of reducing the radiation efficiency by factor 4, being highly significant to the world of radiology and CT scans. This dose reduction allows a safer imaging process for the patient and the medical staff. This method simplifies the system and improves the obtained image quality. The proposed method can contribute additively to standard existing dose reduction or super-resolution techniques to achieve even better performance.

## 1. Introduction

The use of pinhole optics in imaging systems is generally known. The basic principles of a pinhole-based imaging system (e.g., pinhole camera) relate to the direction of radiation/light rays arriving from one point in the object toward a common location on an image plane. This method enables imaging while avoiding the use of refractive lenses, which is replaced by a small aperture. More specifically, light arriving from an object passes through the aperture (small pinhole) and projects an inverted image of the region of interest (the object) on the opposite side of the imaging system. This is also known as the “camera obscura” effect.

Pinhole optics provides several advantages over lens-based optics, such as decreasing linear distortion, providing essentially unlimited depth of focus and wide angular field of view. Moreover, pinhole optics is useful for ionizing radiation, where ordinary lenses become transparent and irrelevant. The above advantages usually have one major con: they reduce the image brightness due to the small diameter of the aperture as compared with the collection area of a lens and reduced resolution due to the finite size of the pinhole [1]. Recently, additional imaging techniques enable using a plurality of pinholes, allowing imaging with increased energy efficiency and proper image restoration using a selected set of pinhole arrays having a suitable arrangement [2,3,4]. The pinholes arrangement enables avoiding loss of data that may result from the superposition of radiation passing through the different pinholes of each array [5,6]. In previous papers, we showed the use of multiple pinholes array in Photon Emission Computed Tomography (SPECT) [7,8]. Using this method, instead of the regular collimator, a sensitivity improvement factor of 5.67 was shown.

In this paper, we would like to expend the use of multi pinholes techniques to more medical modalities such as Computerized Tomography (CT). In the CT procedure, there is a need for a novel configuration and operation technique to enable three-dimensional imaging of a body with decreased radiation level and preferably increasing resolution compared to current conventional CT techniques [9,10,11].

CT is an imaging technique utilizing X-ray imaging from a plurality of angular directions and enabling a three-dimensional mapping of the scanned body. Generally, conventional CT scanning techniques utilize a process of obtaining a plurality of X-ray images. Each of the images relates to input X-ray beam from a different direction and combining the collected data pieces, e.g., by Radon transformation, to generate a three-dimensional model of the inspected body.

CT scans have been a boon for medical care. A CT exam allows physicians to identify internal structures in the human body in high detail. This information can be used to determine if there is a medical problem and reveal other important information that can help the physician to determine the best treatment. CT imaging exams are recognized as a valuable medical tool for a wide variety of examinations and procedures.

Concerns about CT scans include the risks from exposure to ionizing radiation. The relevant biological effect of ionizing radiation is the stochastic effect on the DNA. The main concern is that the DNA may rejoin itself incorrectly with the potential to develop a subsequent malignancy. Although most of the radiation-induced damage is rapidly repaired, disrepair can lead to point mutations, chromosome translocations, and gene fusions that are all linked to cancer induction [12]. This effect is typically thought to be stochastic, and it can occur at any level of radiation exposure, with likelihood increasing as the dose increases [13,14,15].

Several health organizations and regulators call for manufacturers and medical facilities to increase the efforts to minimize this risk by reducing unnecessary exposure to ionizing radiation [16,17].

The proposed method reduces the unnecessary radiation dose drastically without damaging image quality and allows a safe medical imaging process. In the proposed way, the radiation reduction and/or resolution increase is made using minification and a sophisticated encoding of the object on the detector plane by adequately designing the imaging system dimensions and postprocessing.

The benefits of the CT scan are not only for human uses but also for biological research, especially in-vivo diagnostic imaging. The need for this test has increased in several areas such as pathophysiological research and efficient drug screening. However, the radiation dose has the same effect in this field and the reduction need is highly relevant. Alternatively, the proposed method can improve the spatial resolution for better image quality and improve performance [18,19].

There are several methods to improve the spatial resolution of CT systems. Several methods improve the resolution by reducing the physical dimension of clinical CT detectors [20], but, to achieve overall higher spatial resolution with reduced detector size, focal spot size should be reduced accordingly, as shown by Onishi and colleagues [21].

Our proposed technique can also use small physical dimension detectors without the focal spot or the scattering-grid problem because there is no need for such grid in our method. In the regular CT geometry, the scattered radiation should be blocked in front of the detector. In the proposed method, this scattered radiation is also collected by the sensor because our approach is based on imaging instead of projection (projection is very sensitive to focal spot size and to scattered radiation while imaging is not).

Another way to enhance spatial resolution is to improve the sampling done by the detecting units by deflecting the focal spot on the X-ray tube anode along longitudinal and fan angle directions [20] or by using a dual-energy radiation source. This method requires a dual-source CT system in which each X-ray tube produces different X-ray energy spectra, which projects two projection data sets on the detector. Those sets are collected separately for subsequent use in a projection-based dual-energy reconstruction algorithm. The dual-energy radiation source can be used in the proposed method to improve the spatial resolution even more. This issue is out of the scope of this paper and it will be discussed in future work [22].

## 2. Methods

### 2.1. Super-Resolution

Let us assume that the point spread function on the detector plane is notated by *g*(*x*), and the size of each detector in the sampling line is Δ*x*. Then, the readout r(n Δx) (*n* is an integer being the pixel number) equals to:

(1) r(n Δx)=∫Δxs(xM)g(x−nΔx)dx

*s*(*x*/*M*) is our minified imaged object, while *M* is the minification factor. The super-resolution method includes multiple replications with a subpixel shift. Let us assume that we perform *K* replications (*K* is also our aim for the super-resolution factor). Then if we reorder our captured pixels’ readouts, the sampling distance becomes Δx/*K* and thus what we have is:

(2) r(mΔxK)=∫Δxs(xM)g(x−mΔxK)dx

where *m* is an integer.

Mathematically representing discrete samples is done by attaching the values to a train of Dirac delta function. So, we will multiply the discrete series of value by Dirac comb functions:

(3) rs(x)=∑mr(mΔxK)δ(x−mΔxK)=r(x)∑mδ(x−mΔxK)      =(∫Δxs(x′M)g(x′−x)dx′)∑mδ(x−mΔxK)

We will perform a Fourier transform on the last equation:

(4) $$R_{s}\left(\mu \right)=\int^{\;}r_{s}\left(x\right)e^{-i2\pi \mu x}dx$$

(5) S(μM)=∫ s(xM)e−i2πμxdx

(6) $$G\left(\mu \right)=\int^{\;}g\left(x\right)e^{-i2\pi \mu x}dx$$

And obtain:

(7) Rs(μ)=(S(μM)G(−μ))∗(∑mδ(μ−mKΔx))

where ∗ designates convolution operation. Note the integral between minified object *s* and the responsivity *g* is a convolution. The fact that the integral is over the spatial region of Δx is not essential since *g* itself has dimensions that are space limited by Δx. So, it is equivalent to integral from minus infinity to infinity, while the dimensions of *g* are limiting the integral. We will denote:

(8) T(μ)= S(μM)G(−μ)

And obtain:

(9) Rs(μ)=(∑mT(μ−mKΔx))

Thus, one can see from the last two equations that the Fourier transform of those samples equal to replications of the Fourier transforms of the minified object *S* filtered by the Fourier transform of our designed point spread function *G*.

The replications in the spatial frequency domain are distanced with a distance of K/Δx which is *K* time larger from the original sampling distance of 1/Δx. Thus, from the sampling perspective, we have the potential to recover resolution, which is *K* times larger. At that point, the resolution limit is the filter *G*, which should be inverted (inverse, matched, or Wiener filtering). This conversion process is the same method as the aperture encoding in our approach, it makes *G* spectrally wide (the best scenario is where it is *K* times wider than 1/Δx) and invertible. Shaping g within its spatial dimensions of Δx expands its Fourier transform *G* and makes it spectrally wider. By designing *G* well, one can avoid having zeros or low values in the spectrum. All this makes it invertible (like a high rank of a matrix, which makes it more invertible). The more the amplitude of *G* is above zero, the more the function is invertible, and the image signal to noise ratio (SNR) is higher. This filtering process is precisely the purpose of the encoding method and what a robust code aims to have.

The replications and the design of g allow us to have resolution much higher than the original sampling configuration. Since the operation is applied on the minified object, we break the connection between minification factor *M* and resolution. Thus, we can break the relation between radiation dose (related to minification) and spatial resolution. For the optimal case, we also choose *K* = *M* (the minification and the super-resolution factor).

### 2.2. Angular Contrast Noise Ratio (CNR)

CNR is determined by Equation (10) below. The CNR is equal to the difference between the signal and the background divided by the noise’s standard deviation:

(10) $$C=\frac{\left|S_{A}-S_{B}\right|}{\sigma_{N}}$$

We assume that the aim is to preserve the same amount of noise while improving resolution (alternatively, one can also maintain the exact resolution and improve the SNR). For this case, we will approximate and use Parseval relation (energy in space domain and Fourier domain is conserved since the Fourier transform is an energy-conserving transform):

(11) $$C\approx \frac{S_{A}}{\sigma_{N}}-\frac{S_{B}}{\sigma_{N}}=\frac{\int_{\Delta \mu_{s}}MTF\left(\mu \right)d\mu }{\sigma_{N}}-\frac{\int_{\Delta \mu_{b}}MTF\left(\mu \right)d\mu }{\sigma_{N}}$$

The approximation is for the logical assumption of $S_{A}>S_{B}$, i.e., the signal is stronger than the background. In the last Equation (11) $\Delta \mu_{s}$ is being the spectral bandwidth of the signal while $\Delta \mu_{b}$ is the spectral bandwidth of the background. The improvement factor in *C* depends on the super-resolution factor.

One can mark the CNR without the above super-resolution process as *C*_0_, thus the *C_SR_*, i.e., the CNR with the super-resolution factor of *K* can approximately be:

(12) $$\begin{array}{l} C_{SR}=\frac{\int_{K\Delta \mu_{s}}MTF\left(\mu \right)d\mu }{\sigma_{N}}-\frac{\int_{\Delta \mu_{b}}MTF\left(\mu \right)d\mu }{\sigma_{N}}\approx \frac{K\int_{\Delta \mu_{s}}MTF\left(\mu \right)d\mu }{\sigma_{N}}-\frac{\int_{\Delta \mu_{b}}MTF\left(\mu \right)d\mu }{\sigma_{N}}\\ \;\;\;\;\;\;=\frac{\left(K-1\right)\int_{\Delta \mu_{s}}MTF\left(\mu \right)d\mu }{\sigma_{N}}+\frac{\int_{\Delta \mu_{s}}MTF\left(\mu \right)d\mu }{\sigma_{N}}-\frac{\int_{\Delta \mu_{b}}MTF\left(\mu \right)d\mu }{\sigma_{N}}\\ \;\;\;\;\;\;=\frac{\left(K-1\right)\int_{\Delta \mu_{s}}MTF\left(\mu \right)d\mu }{\sigma_{N}}+C_{0} \end{array}$$

Thus, as *K* gets larger than 1, the larger is the improvement in the CNR obtained.

### 2.3. Fisher Information Matrix

The inverse of the Fisher information matrix, *I*(*θ*), provides a lower bound for the variance of an unbiased estimator ($\hat{\theta }$), or especially, the Cramer–Rao lower bound, $var\left(\hat{\theta }\right)\geq I^{-1}\left(\theta \right)$ [23]. If this fundamental lower bound can be calculated for a given photon distribution (which is just the point spread function (PSF) response of the instrument in question), then the localization limit can be derived; this localization limit is given by $\sqrt{I^{-1}\left(\theta \right)}$ where the Fisher information matrix *I*(*θ*), is calculated as [24]:

(13) $$\begin{array}{l} I\left(\theta \right)=E\left\{{\left(\frac{\partial }{\partial \theta }log\left(f\left(X;\theta \right)\right)\right)}^{2}|\theta \right\}\\ \;\;\;\;\;\;=\gamma A\left(\Delta t\right)\underset{{ℜ}^{2}}{\int }\frac{1}{g\left(x,y\right)}{\left[\frac{\partial g\left(x,y\right)}{\partial x},\frac{\partial g\left(x,y\right)}{\partial y}\right]}^{T}\left[\frac{\partial g\left(x,y\right)}{\partial x},\frac{\partial g\left(x,y\right)}{\partial y}\right]dxdy \end{array}$$

where *f* is the probability density function, and *X* is the observable random variable that carries information about the unknown variable *θ* which we aim to extract. *E*{…} is the average ensemble operator. *γ* is the imaging system’s detection efficiency, *A* is the emission rate of the point source generating the photon distribution, and Δ*t* is the recording/integration time. *g*(*x*, *y*) is the PSF of the imaging system.

Thus, the product *γA*Δ*t* yields the total number *N* of detected photons or the sampling density for the given distribution. Furthermore, if the PSF in question is symmetric, then the off-diagonal elements of the Fisher information matrix go to zero (since it is a tensor operator), and the two diagonal elements are equal, simplifying the calculation. For a PSF of *g*(*x*, *y*) given by, e.g., a Gaussian distribution having spatial width (STD of the spatial Gaussian) denoted by Δ*x*, this will lead to:

(14) I(θ)=NΔx2

From the last equation, one can estimate the width of the PSF (localization or a resolution of a single PSF), which is equal to:

(15) Δx^=I−1(θ)=ΔxN

One can see that in a case where the SNR remains the same, and the resolution has improved, the number of photons, *N*, remains the same regardless of the super-resolution process. Still the PSF’s width becomes narrow, i.e., the ∆*x* becomes *K* times smaller by the super-resolution process (*K* is the super-resolution factor). Then, a ratio Θ can be taken while this ratio yields the improvement in the localization accuracy for the modified super-resolved PSF compared to the regular Gaussian-like PSF, for the case when the sampling density, i.e., the number of the collected photons is the same. Namely,

(16) $$\Theta =\sqrt{\frac{{I^{-1}}_{SR\;PSF}\left(\theta \right)}{{I^{-1}}_{Gaussian\;PSF}\left(\theta \right)}}=\frac{\Delta x_{SR\;PSF}}{\Delta x_{Gaussian\;PSF}}\approx \frac{1}{K}$$

This derivation shows that when the SNR is preserved, and super-resolution of factor *K* is obtained, also according to Fisher information matrix, one obtains an improvement by a factor of *K*. Obviously, the same goes for the other case when the resolution is preserved, while the SNR is improved due to the minification factor.

Thus, if we break the bound between resolution and minification, we can, as explained above, have the same resolution but to have a minification factor of *K*, which means that for the exact number of pixels in the detector, we have $K^{2}$ times more photons (the imaging system is two-dimensional, including the detector). If we refer again to the Fisher information matrix expression that was derived above, then we obtain:

(17) Δx^=I−1(θ)=ΔxK2N

which means that the SNR improvement factor will be:

(18) Θ=I−1SR PSF(θ)I−1Gaussian PSF(θ)=ΔxK2NΔxN≈1K

### 2.4. Angular Improvement Via Depth Resolution

Due to the minification, the angular range that needs to be scanned is reduced by the factor *M* well. As stated earlier, with the super-resolution proof, the main concern is whether the reduction in the angular range will damage the obtainable 2D resolution (for a slice reconstruction).

The depth resolution obtained in our case is similar to the triangulation relation, and even from a single view, depth information can be extracted. The depth information equals to:

(19) $$Z=F\frac{D}{X{}^{\prime }}$$

where *D* is the diameter of our holes array plate or, more precisely, it is the distance between two external holes in the holes array plate. *X*’ is the disparity along the *X* coordinate in imaging the same point in the object, *F* is the focal length of our imager, which is the distance between the detector and the holes array plate. *Z* is the distance between the holes array plate and a given point in the object. Thus, by performing a derivation of the last equation according to *X*’, one can obtain the depth resolution obtainable from a single image:

(20) $$\left(dZ\right)=F\frac{D}{{X^{\prime }}^{2}}\left(dX{}^{\prime }\right)$$

where *dZ* and *dX’* are the resolution obtained in *Z* (depth) and along the *X* axis, respectively. By substituting the last two equations into each other, one obtains:

(21) $$\left(dZ\right)=\frac{Z}{F}\times \frac{Z}{D}\times \left(dX{}^{\prime }\right)=M\times \frac{1}{tan\theta }\times \left(dX{}^{\prime }\right)$$

while *M* is the magnification factor and *θ* is the angle formed by a single point in the object and the two external holes in the array.

Let us assume that the proposed configuration is designed such that the angular resolution obtained in a single view of the proposed imaging concept is at least as good as:

(22) (The angular resolution of regular CT)×(The minification factor M)

In this case, to produce the same 3D reconstruction, the system needs M times fewer viewing angles. The minification factor *M*, which is also the field/region of interest (ROI) reduction factor, is the reduction factor in the angular scanning process, to allow the same depth resolution (as in regular CT). The principal conclusion is that the total reduction in the dose will be equal to *M*^3^ (since an additional *M* factor also reduced the angular ROI that equals *M*^2^ factor as shown in the previous sections above).

### 2.5. Multiplexing and Encoding Effect

Let us assume that we have an additive noise *n* added to our signal *s*, and *T* denotes the mixing (multiplexing matrix of our encoding). In this case, the measured output equals:

(23) $$y=T\left\{s+n\right\}=Ts+Tn≜y_{s}+y_{n}$$

To reconstruct our signal *s*, one needs to invert the mixing matrix *T*, where *y_s_* is the part of the signal, and *y_n_* is the part of the noise.

If there had been no mixing (*T* is an identity matrix *I*), the SNR would have been:

(24) $${\mathrm{SNR}}_{No\;mixing}=\frac{\sum_{i=1}^{N}\left|{\lambda_{i}}^{\left(s\right)}\right|}{{\sigma_{n}}^{2}}$$

where ${\sigma_{n}}^{2}$ is the covariance of the noise *n* and according to what we have proved above $\overset{N}{\underset{i=1}{\sum }}\left|{\lambda_{i}}^{\left(s\right)}\right|$ is the energy of the signal. According to what we showed, it also equals to:

(25) $${\mathrm{SNR}}_{No\;mixing}=\frac{\sum_{i=1}^{N}\left|{\lambda_{i}}^{\left(s\right)}\right|}{\sum_{i=1}^{N}\left|{\lambda_{i}}^{\left(n\right)}\right|}$$

where ${\lambda_{i}}^{\left(s\right)}$ are the eigenvalues of the signal *s* and ${\lambda_{i}}^{\left(n\right)}$ are the eigenvalues of the noise *n*.

Due to the mixing/multiplexing, we get the covariance matrix equal to:

(26) $$\Lambda_{tot}=E\left\{\left[\begin{array}{c} T^{-1}y_{s}\\ T^{-1}y_{n} \end{array}\right]\left[\begin{array}{cc} {\left(T^{-1}y_{s}\right)}^{T} & {\left(T^{-1}y_{n}\right)}^{T} \end{array}\right]\right\}\;\;\;\;\;\;\;\;\;\;\;\;\;\;\;\;=\left[\begin{array}{cc} T^{-1}E\left\{{y_{s}}^{T}y_{s}\right\}{\left(T^{-1}\right)}^{T} & T^{-1}E\left\{{y_{n}}^{T}y_{s}\right\}{\left(T^{-1}\right)}^{T}\\ T^{-1}E\left\{{y_{s}}^{T}y_{n}\right\}{\left(T^{-1}\right)}^{T} & T^{-1}E\left\{{y_{n}}^{T}y_{n}\right\}{\left(T^{-1}\right)}^{T} \end{array}\right]$$

Since the signal and the noise are independent of each other and the noise has zero mean, we get:

(27) $$\Lambda_{tot}=\left[\begin{array}{cc} T^{-1}\left(\overset{N}{\underset{i=1}{\sum }}\left|{\lambda_{i}}^{\left(s\right)}\right|\right){\left(T^{-1}\right)}^{T} & 0\\ 0 & T^{-1}\left(\overset{N}{\underset{i=1}{\sum }}\left|{\lambda_{i}}^{\left(n\right)}\right|\right){\left(T^{-1}\right)}^{T} \end{array}\right]$$

Thus, the SNR to be obtainable will be:

(28) $${\mathrm{SNR}}_{Mixing}=\frac{\frac{adj\left(T\right)adj{\left(T\right)}^{T}}{{\left(det\left(T\right)\right)}^{2}}\sum_{i=1}^{N}\left|{\lambda_{i}}^{\left(s\right)}\right|}{\frac{adj\left(T\right)adj{\left(T\right)}^{T}}{{\left(det\left(T\right)\right)}^{2}}\sum_{i=1}^{N}\left|{\lambda_{i}}^{\left(n\right)}\right|}=\frac{\frac{\sum_{i=1}^{N}\left|{\lambda_{i}}^{\left(T\right)}\right|}{{\left(det\left(T\right)\right)}^{2}}\sum_{i=1}^{N}\left|{\lambda_{i}}^{\left(s\right)}\right|}{\frac{\sum_{i=1}^{N}\left|{\lambda_{i}}^{\left(T\right)}\right|}{{\left(det\left(T\right)\right)}^{2}}\sum_{i=1}^{N}\left|{\lambda_{i}}^{\left(n\right)}\right|}\;\;\;\;\;\;\;\approx \frac{\frac{1}{{\left(det\left(T\right)\right)}^{2}}\sum_{i=1}^{N}\left|{\lambda_{i}}^{\left(s\right)}\right|}{\frac{1}{{\left(det\left(T\right)\right)}^{2}}\sum_{i=1}^{N}\left|{\lambda_{i}}^{\left(n\right)}\right|}$$

where $\lambda^{\left(T\right)}$ are the eigenvalues of the mixing/multiplexing matrix *T*. This leads to:

(29) $${\mathrm{SNR}}_{Mixing}\approx \frac{\sum_{i=1}^{N}\left|{\lambda_{i}}^{\left(s\right)}\right|}{\sum_{i=1}^{N}\left|{\lambda_{i}}^{\left(n\right)}\right|}={\mathrm{SNR}}_{No\;mixing}$$

Theoretically, as can be seen from the last equation, the SNR is not damaged by the mixing process, since the same value was added in the nominator and the denominator. The SNR is not damaged except the case where the mixing is invertible; otherwise, it will damage the SNR [25]. The problem occurs for the cases where the electronic noise due to insufficient number of bits at the A/D adds quantization noise such that for given spatial frequencies where the signal is low, the $1/{\left(det\left(T\right)\right)}^{2}$ expression, which is needed for the multiplexing inversion process (*T*^−1^) causes noise amplification (while the signal is not amplified since it is already distorted due to the quantization process). However, in a case where the A/D convertor in the detector has a sufficient number of quantization bits and it is sufficiently accurate, it allows the SNR to be treated as analog information. In this case, the multiplexing procedure is completely invertible, which leads to the same SNR as in the case where no multiplexing was done.

Thus, for the proposed method to act well, two components are needed: (1). Robust multiplexing code which means that it is highly invertible code, which means that det(T) is not too small. (2). The A/D of the detector has a large number of quantization bits that will not add considerable quantization noise and will not distort the SNR also for spatial frequencies in which the SNR is low.

This fundamental problem can also be explained in voxel terminology: if one assumes that there is the more or less similar spatial density of X-rays passing through the object, then by performing a minification by a factor of *M* we increase the voxel by the same factor and thus we have *M*^2^ more rays passing through it. In the conventional case, increasing the voxel in the object also destroys resolution by *M*^2^ (the same factor by which the number of rays is increased). 

We show here that despite the increase of the voxel by *M*^2^, we preserve the same resolution. Thus, we show that we have *M*^2^ more rays passing through the same voxel of resolution without damaging the reconstruction resolution. The above means that we can reduce the irradiation dose by an *M*^2^ factor and then have after the reduction effectively the same number of rays passing through the same voxel of resolution.

## 3. Results

### 3.1. Simulations

The simulations were done using Geant4 10.3 [26,27,28] toolkit for Monte-Carlo, high-energy particle transport. This software is a public-domain software package composed of tools that can simulate the passage of particles through matter accurately.

**Figure 1:**
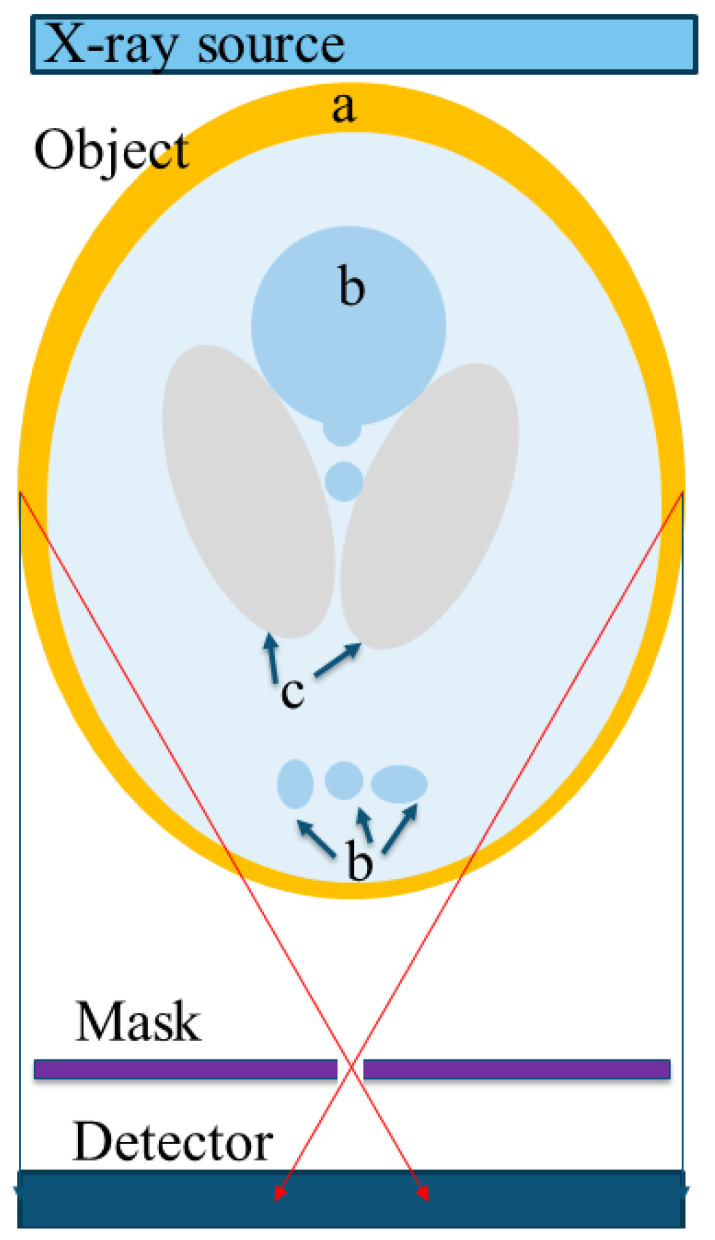
The simulation setup consists of an X-ray source, an object (a standard phantom or any other shape), a filter coding mask, and a flat panel detector. In this figure, the object is a Shepp–Logan Phantom, a standard test object of a head section which is composed of: (**a**) skull, (**b**) glandular tissue, and (**c**) soft tissue. The red arrows represent two radiation rays passing through one of the pinholes in the mask.

The simulation was mainly composed of four parts: X-ray source, object, pinholes mask, and detector, as shown in Figure 1. One of the benefits stems from the fact that the system is based on imaging instead of projection in that there is no need for antiscattering grid in front of the detector. The system utilizes the scatterings as additional sources within the object and thus increases the imaging system’s effectiveness. Removing the antiscattering grid also simplifies the design mechanically and allows using flat panel detectors with smaller pixels.

The particle sources were simulated using the Geant4 General Particle Source (GPS), which is part of the Geant4 toolkit. Specifically, it allows the specifications of the spectral, spatial, and angular distribution of the particle source. We use a set of X-ray particle point sources with an energy of 100 KeV. The angular distribution is confined to the pinhole’s location.

Radiation rays, or photon beams, are characterized by their intensity, the number of photons, and their energy, limited by the incident electron’s energy, which is equal to the voltage on the tube times the electron charge. The attenuation coefficient measures the probability of the interaction between incident photons and the matter of the unit mass per unit area. In a monoenergetic photon beam, the attenuation coefficient is constant. In contrast, in a polyenergetic photon beam, lower energies have a higher probability for interactions, and they attenuated more rapidly than higher energies. Therefore, the average energy of the photon beam increases with attenuation. This effect is known as filtration or beam hardening [29]. This effect causes the object’s edges to appear brighter than its center, even if the material is the same throughout. The simulation performed with monoenergetic 100 keV ray, so there is no beam hardening effect or correction in the process. Still, the effect is the same in the proposed method and so is the correction method. There are several beam hardening correction (BHC) methods and studies [30,31,32,33], and most of those methods can be integrated into the proposed system. In the experimental Section 3.2 below, where a 60 kVp tube has been used, the beam hardening was corrected by a calibration process and linear correction method [29,30,31,32,33].

The object was a modified Shepp–Logan phantom [34]. This phantom was composed of several ellipses made of different materials. The materials were defined using Geant4 advanced example of a human phantom, according to [35]. In the simulation, we used MIRD skeleton, MIRD glandular, and MIRD lung. The pinholes canonic shape is done by defining Geant4 Box made of tungsten and adding Geant4 Elliptical Cones made of vacuum in the pinhole’s location. The detection was done by a Geant4 sensitive detector, which counts every particle entrance to the detector pixels during the event action.

Our simulation used GNU Parallel [10], a shell tool for executing parallel jobs. The radiation source in the simulation was divided into several separate ray-tracing simulations where each X-ray source runs individually; this process shortens the simulation running time.

The simulation results were obtained for ×0.25 magnification, a detector pixel size of 0.13 mm, and 375 pixels. The magnification factor depends on the ratio between two distances: object to the mask and mask to the detector.

We compared our results to the current techniques by a simulation of parallel beam CT geometry with ×1 magnification, a detector pixel size of 0.13 mm, and 1500 pixels. The exposure time (e.g., radiation dose) for the parallel beam geometry was 16 times higher than the exposure time for the proposed geometry. However, in the proposed geometry four images were taken for each view. Each image was taken with 0.25 pixel shift of the detector. This shift enables reconstruct of the same pixel resolution of 1500 pixels in our geometry by using the super-resolution technique, as explained in detail in the methods section. A summary of the parameters is given in Table 1.

The simulations were done for two different scenarios: the same rays per view for the current and the proposed methods and ×4 rays for the existing geometry. For each scenario, the signal to noise ratio (SNR) and the contrast to noise ratio (CNR) were calculated using the equations:

(30) $$\mathrm{SNR}=\frac{mean}{std}$$

where *mean* is the mean pixels value over the selected area and *std* is the standard deviation of these pixels.

(31) $$\mathrm{CNR}=\frac{mean_{1}-mean_{2}}{\sqrt{std_{1}+std_{2}}}$$

where indices 1 and 2 refer to two adjacent areas.

**Figure 2:**
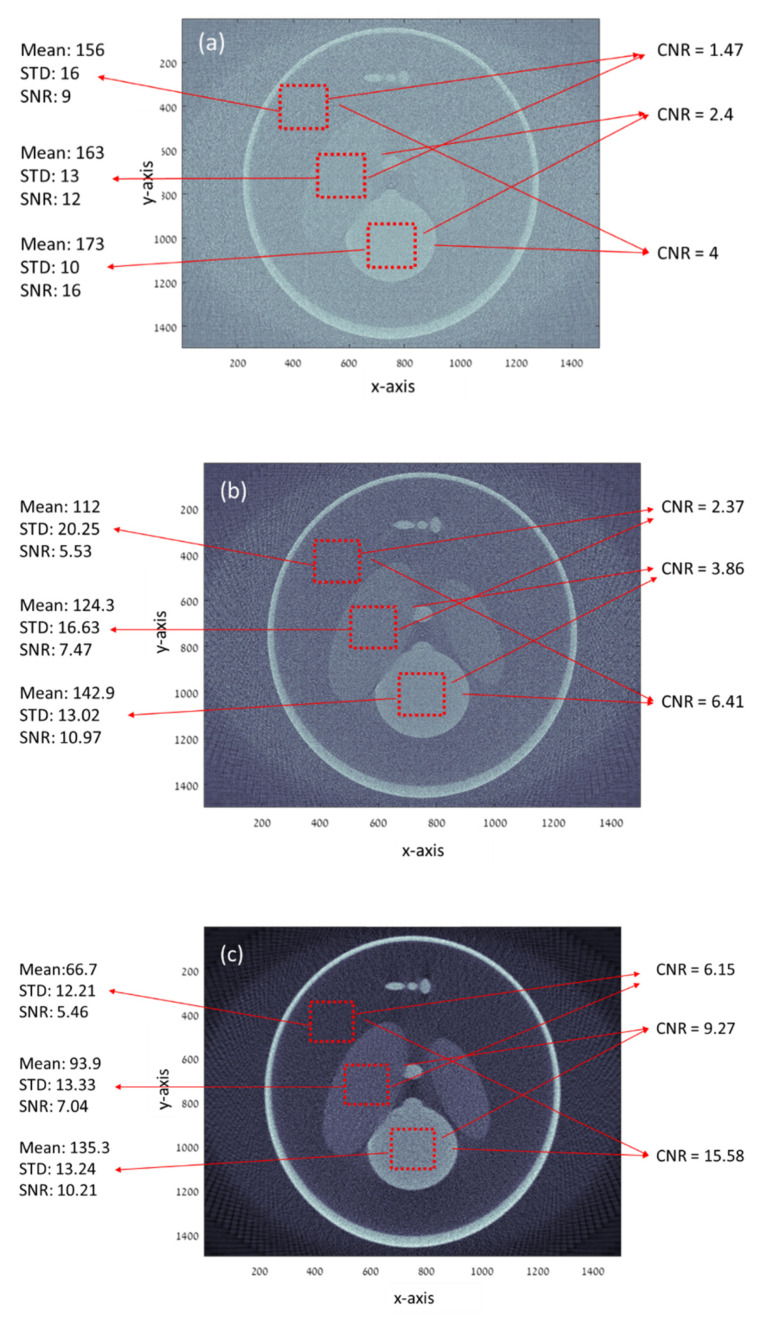
Simulation results of the modified Shepp–Logan phantom for the current CT geometry with different dose represented by the number of radiation rays: (**a**) 750,000 rays per view and (**b**) 3,000,000 rays per view and (**c**) the results for the proposed geometry with 750,000 rays per view (4 images of 187,500 rays per view) that corresponds to the number of rays in (**a**).

Figure 2 presents the obtained reconstructed images for 180°, with an image at each degree for current geometry and proposed geometry. The 2D image of each view can be reconstructed using inverse filtering. However, the inverse filtering technique has a major disadvantage: noise amplification. Therefore, other algorithms can be applied specifically to each application, e.g., matched filter, Wiener filter, adaptive wiener filter, Tikhonov regularization, Richardson–Lucy algorithm, etc. [36,37,38,39].

In our reconstruction process, the 2D image reconstruction was done using the Wiener filter [40]:

(32) $$H=\frac{G^{*}}{G\times G^{*}+h}$$

where *G* is the point spread function of the system, $G^{*}$ is the adjacent complex of *G*, and *h* is the noise reduction factor.

After applying the Wiener filter for each view, every image was rotated according to the acquisition angle. All the images were summed up and filtered to obtain a complete reconstruction of a slice, as shown in Figure 2.

**Figure 3:**
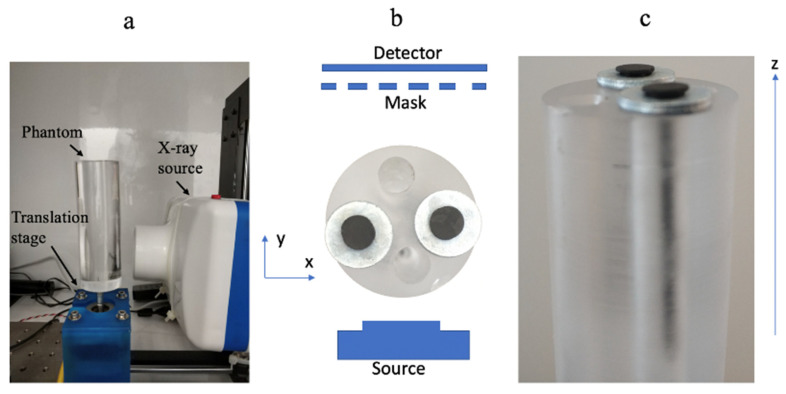
(**a**) The experimental setup: the source, and the phantom are shown. (**b**) Schematic sketch of the experimental configuration with the flat panel detector and the coding mask. (**c**) The object included two metallic nails in the cylindric polymer.

One can see in Figure 2c and Figure 3a that CNR improved by about 400% when both geometries had the same radiation and there was an almost 250% CNR improvement when the proposed method had less radiation dose by factor 4. The SNR in Figure 2c,d is about the same. This fact indicates that the proposed method can improve the CNR at less radiation and preserve the SNR. One should notice the high SNR in Figure 2a. This high SNR stems from the very low resolution since SNR is inversely proportional to the basic resolution [41,42].

A summary of the simulation results is given in Table 2.

### 3.2. Preliminary Experimental Results

The setup consists of a phantom, a uniform acrylic rod with 27 mm diameter, and 2 metallic nails inside it. Each nail is 2.5 mm diameter. The setup also includes a multihole mask, an X-ray source (Dental BLX-5, 60 kVp, TIANJIE, Zhengzhou, China), a self-constructed *XYZ* motorized stage, and a translation stage with stepper motor and a detector (CCD Data acquisition module, Hamamatsu Photonics, Shizuoka, Japan), as shown in Figure 3.

**Figure 4:**
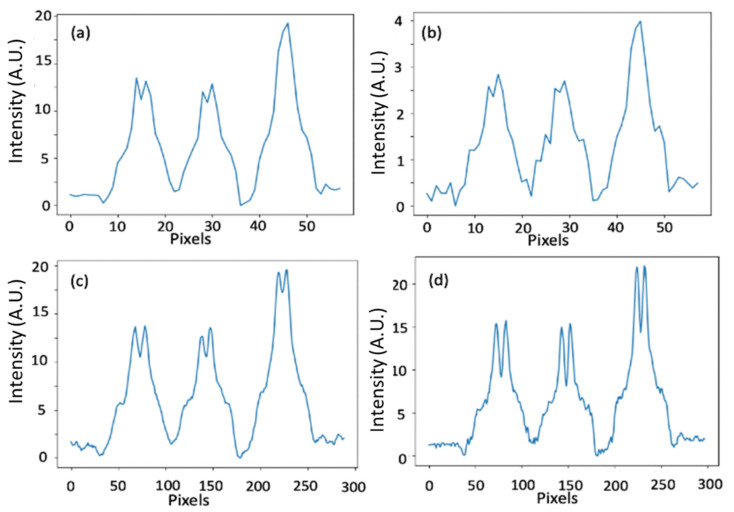
A single view’s projection. (**a**,**b**) are the low-resolution projections with an exposure time of *T* and *T*/5, respectively, the pixel size is 0.5 mm. (**c**) The high-resolution reconstructed image. (**d**) A high-resolution reference projection was obtained with a pixel size of 0.1 mm and an exposure time of *T*.

In Figure 4 we present a single view projection.

Figure 4a shows regular detector data. There the detector pixel size was 0.5 mm, and the exposure time was *T*. In Figure 4b, we present one of five projection sets done from the same single view, each projection was taken with a different mask at 0.2 pixels shift (1/5 of a pixel = 0.1 mm). The detector pixel size was 0.5 mm, and the exposure time was *T*/5. Figure 4b is the same image as Figure 4a, but it is noisier as it has five times shorter exposure time. Figure 4c shows the summation of five images of Figure 4b with a 0.2 pixel shift. The effective pixel size was 0.5 mm and the exposure time is (*T*/5) × 5 = *T*. Figure 4c is the created high-resolution projection that we generated with our super-resolving concept. Figure 4d shows the high-resolution projection obtained with a pixel size of 0.1 mm and exposure time of *T*. We use it as a reference. From the projection images presented in Figure 4, one may see that indeed the resolution in the images of Figure 4a,b is low. In contrast, the resolution of the reconstructed projection of Figure 4c resembles the high-resolution reference of Figure 4d.

**Figure 5:**
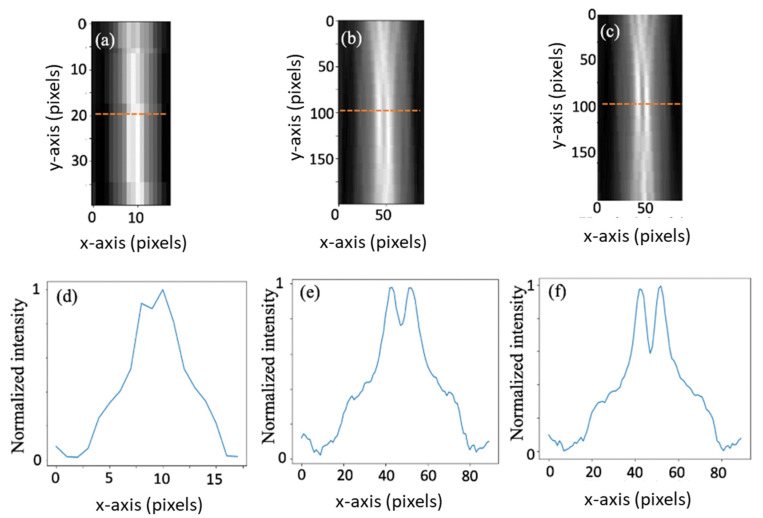
A single view reconstruction. (**a**,**b**,**c**) are reconstructions of a slice from a single view where (**a**) is a low-resolution image, (**b**) is a high-resolution image using our super-resolution concept, and (**c**) a high-resolution reference image. (**d**,**e**) and (**f**) are intensity profiles of the cross-sections marked with red lines in (**a**,**b**) and (**c**), respectively.

In Figure 5, we show the reconstruction slice from a single view.

In Figure 5a one can see reconstruction from the low-resolution projection of Figure 4b, in Figure 5b the reconstruction created from the high-resolution projection of Figure 4c, and in Figure 5c the reconstruction made from the reference projection of Figure 4d. If we compute the correlation coefficient (the Pearson product-moment correlation coefficients) between the high-resolution reference of Figure 5d and the high-resolution reconstruction of Figure 5c, we obtain a coefficient of 0.97 for the region of the two nails. The above means that, indeed, the reconstructed high-resolution image was able to capture almost the same spatial information as contained within the high-resolution reference image. If, on the other hand, we compute the corresponding correlation coefficient between the low-resolution image of Figure 4a, we have a coefficient of only 0.75.

## 4. Discussion

The resolution conservation with less radiation can also be explained in voxel terminology: if one assumes that there is, more or less, a similar spatial density of X-rays passing through the object, then by performing a minification by a factor *M* we increase the voxel by the same factor and thus we have *M*^2^ more rays passing through it. In the conventional case, increasing the voxel in the object also destroys resolution by a factor of *M*^2^ (the same factor by which the number of rays is increased).

Despite the voxel increase by *M*^2^, the resolution was preserved. Thus, one can see that without damaging resolution of reconstruction, we have *M*^2^ more rays passing through the same voxel of resolution. This focusing process means that one can reduce the irradiation dose by an *M*^2^ factor and then have, after the reduction, effectively the same number of rays passing through the same voxel of resolution.

Moreover, the proposed method also reduces the number of images required to reconstruct a three-dimensional model of the monitored object or body. More specifically, the proposed method enables the obtaining of three-dimensional data using image data collected from a single angular direction, a view. This reduction of view angles stems from the pinhole arrays’ different locations providing a slightly different point of view of the inspected object, thereby effectively providing stereoscopic imaging. Using three-dimensional image reconstructions for each angular direction enables the proposed system to provide a high-resolution three-dimensional reconstruction of an object while requiring a reduced number of images as compared to conventional projection-based CT systems. This reduction in the necessary images enables the proposed method to provide high-resolution three-dimensional modeling of body parts or organs while reducing the required radiation.

Additionally, since the detector uses a smaller number of pixels, it can be inferred that the total computational power will also be reduced with the same factor *M*. Despite the requirement for more images per view, for the super-resolution process, the algorithm is using fewer views to create the same sinogram as the current geometry produces.

Furthermore, the proposed technique is appropriate for imaging with optical and nonoptical spectral ranges. It is incredibly valuable for imaging with nonoptical spectral ranges because of the inability to use regular lenses in those ranges. These nonoptical wavelength ranges can include X-ray, Gamma, UV, alongside ultra-sound and other wave-like radiation.

## 5. Conclusions

This paper showed simulation and preliminary experimental validation for reducing the irradiation dose using super-resolving aperture-coded imaging concept. It was shown that if a minification factor of *M* is used in our enhanced reconstruction, the energy concertation per the same area of a pixel will be increased by a factor of *M*^2^. However, in the conventional case, the resolution will also be destroyed by the same factor.

Thus, we showed an experimental validation in which, even though we perform a minification (in this case by a factor of *M* = 5), the resolution is almost not damaged. Thus, we manage to break the connection between energy concentration and resolution, which directly shows the reduction in the irradiation dose. This means that we can reduce the irradiation dose by a factor of *M*^2^ and then have effectively the same number of rays passing through the same voxel of resolution. In other words, we showed that despite the increase of the voxel by *M*^2^, we preserve the same resolution. Thus, we show that without damaging the reconstruction resolution, we have *M*^2^ more rays passing through the same voxel of resolution. This dramatic dose reduction allows a safer imaging process for the patient and for the medical staff. It also simplifies the system, allows a smaller pixel size without an antiscattering grid, and reduces the required postprocessing computing power. The proposed method can be used additively to other existing dose reduction methods or super-resolution techniques to achieve even better performance.

## 6. Patents

This work is protected by: [43,44,45].

## Figures and Tables

**Table 1:** Simulation’s parameters summary.

Parameter	Current Geometry	Proposed Method	Proposed Method after Super-Resolution
Detector resolution	1500 pixels	375 pixels	1500 pixels
Pixel size	0.13 mm	0.13 mm	0.0325 mm
Exposure time	*T* and *T*/4	*T*/16	*T*/4
Number of images	1	4	1

**Table 2:** Simulation’s results summary.

Parameter	Current Geometry	Current Geometry	Proposed Method
Rays per view	3,000,000	750,000	750,000
SNR1	5.53	9	5.46
SNR2	7.47	12	7.04
SNR3	10.97	16	10.21
CNR12	2.37	1.47	6.15
CNR23	3.86	2.4	9.27
CNR13	6.41	4	15.58
